# Rosiglitazone Add-On in Treatment of Depressed Patients with Insulin Resistance: a Pilot Study

**DOI:** 10.1100/tsw.2010.32

**Published:** 2010-02-19

**Authors:** Natalie L. Rasgon, Heather A. Kenna, Katherine E. Williams, Bevin Powers, Tonita Wroolie, Alan F. Schatzberg

**Affiliations:** ^1^ Stanford Center for Neuroscience in Women's Health, Stanford University School of Medicine, Stanford, CA, USA; ^2^ Mood Disorders Program, Department of Psychiatry and Behavioral Sciences, Stanford University School of Medicine, Stanford, CA, USA

**Keywords:** insulin resistance, depression, Matsuda Index, rosiglitazone

## Abstract

A number of cross-sectional studies have suggested an association between insulin resistance (IR) and affective disorders. However, limited data exist on potential changes in IR in a prospective treatment of depression. The present pilot study tested the hypothesis that improvement of IR with the addition of an insulin-sensitizing agent would improve mood in nondiabetic patients with unipolar or bipolar depression, who had surrogate blood markers suggestive of IR. Surrogate IR-criteria blood markers were fasting plasma glucose >100 mg/dl or triglyceride (TG) to high density lipoprotein (HDL) ratio >3.0. Open-label rosiglitazone, titrated to a dose of 8 mg/day, was administered for 12 weeks to 12 patients with depressive disorder receiving treatment as usual (TAU). Eight patients who completed the 12-week study exhibited significant declines in both depression severity by the Hamilton Depression Rating Scale and the Clinical Global Impression scale, with moderate effect sizes noted. Modest improvement in Matsuda Index scores was also noted at 12 weeks, yet declines in depression severity scores were not associated with improvements in the endocrine markers (Matsuda Index, TG/HDL ratio, and body mass index). These results suggest the potential novel use for an insulin-sensitizing agent in the treatment of depressive disorders. Larger placebo-controlled studies are warranted.

## INTRODUCTION

An association between insulin resistance (IR) and affective disorders has been postulated in a number of cross-sectional studies[1,2,3,4,5,6,7,8,9,10,11,12,13,14,15,16,17]. Limited data exist on potential changes in IR associated with improvement in depressive symptoms and/or depression remission resolution – two studies reported decreased IR after successful antidepressant treatment[1,18], while another study reported persisting IR even after successful treatment[15].

We have postulated that IR is a part of the pathophysiology of affective disorders, and its improvement (via pharmacological or nonpharmacological treatments) may significantly reduce the severity of depressive symptoms[19,20]. In support of this hypothesis, we previously reported increased IR in women with bipolar disorder[14,21,22,23], as well as a significant association between IR and depressive symptoms in women with primary IR syndrome (polycystic ovary syndrome [PCOS])[24,25].

In the current pilot study, we attempted a more direct testing of the hypothesis that improvement of IR will result in improvement in mood in patients with depressive disorders. The aim of the study was to evaluate whether addition of the peroxisome proliferator-activated receptor-γ (PPAR) agonist rosiglitazone to the treatment as usual (TAU) of nondiabetic patients with unipolar or bipolar depression would result in improvement in depression severity and clinical global impression (CGI). Insulin-sensitizing agents have proven efficacious in nondiabetic IR, or “prediabetic”, individuals[26]. All subjects in this pilot study had elevated fasting plasma glucose (FPG) and ratios of high density lipoproteins (HDL) to triglycerides (TG), which are established surrogate markers of IR[27,28].

## METHODS

The study was approved in its entirety by the Stanford University Administrative Panel on Human Subjects. After providing verbal and written informed consent, patients underwent the screening process, which included the Structured Clinical Interview for DSM-IV (SCID), clinician rating on the 21-item Hamilton Depression Rating Scale (HDRS-21) and the Clinical Global Impression-Severity scale (CGI-S), measurement of height, vital signs (including weight), and fasting blood sampling for plasma glucose, TG, and HDL. Hepatic function was also assessed at screening, midtreatment, and end of treatment to ensure patient safety. Inclusion criteria for the study included formal diagnosis for Major Depressive Disorder or Bipolar Disorder-Current Episode Depressed confirmed by both the patient’s mental health treatment provider and on clinical interview with the SCID, residual depressive symptoms as indicated by a score ≥12, and indication of nondiabetic IR, as demonstrated by a surrogate biomarker (FPG ≥ 100 mg/dl or TG/HDL ≥ 3.0). FPG ≥ 100 mg/dl is a well-accepted cut-off for indication of glucose intolerance[29], and TG/HDL ratio has been proven to be a highly sensitive surrogate biomarker of IR in nondiabetic individuals[28]. Exclusion criteria included type 2 diabetes, as indicated by FPG ≥ 120 mg/dl[29].

Prior to initiation of rosiglitazone treatment, all patients underwent assessment of their glucose utilization by a 2-h 75-mg oral glucose tolerance test (OGTT), with blood samples drawn every 30 min (T0, T30, T60, T90, and T120 minutes) for both plasma glucose and insulin. The OGTT was subsequently repeated at the end of the 12-week treatment, as was measurement of TG/HDL ratio. The Matsuda Index for insulin sensitivity was calculated from both baseline and 12-week OGTT data using the standard formula[30], with higher index scores indicating less IR. Body mass index (BMI), as assessed by height and weight in $kg/m^2$, was measured at screening and at each study visit. This was a pilot study and, as such, was not powered for determining any effect size, but rather just to evaluate prospectively the antidepressive effects of a PPAR-agonist add-on medication to TAU. Further, as the study was conceived prior to the negative meta-analysis on rosiglitazone[31], which has been since disputed[32], we utilized this PPAR-agonist in all subjects, rather than switching to another thiazolidinedionespioglitazone, such as pioglitazone. All subjects were advised about potential side effects of rosiglitazone prior to study entry and again after the negative review of the literature was published[31].

Rosiglitazone was administered in addition to current antidepressant and/or mood-stabilizing medication at a dose of 4 mg/day for the first 4 weeks, with subsequent increase in dose to 8 mg/day for the remaining 8 weeks of the 12-week trial. Vital signs (including weight) and adverse events were monitored throughout the study at 3-week intervals, and a hepatic functioning was assessed at treatment weeks 6 and 12 to ensure patient safety. Depression severity was assessed with the HDRS-21 and CGI-S

at baseline and treatment weeks 6 and 12. Table 1 describes the concurrent psychotropic medications used by subjects in this study.

**TABLE 1**
**Concurrent Medications**

| | |
|---|---|
| **Study Completers** | |
| 1 | Nortriptyline (75 mg), lithium (300 mg), lamotrigine (100 mg) |
| 2 | Paroxetine (40 mg), lamotrigine (150 mg) |
| 3 | Sertraline (125 mg) |
| 4 | Citalopram (20 mg) |
| 5 | Duloxetine (60 mg) |
| 6 | Adderrall (60 mg) |
| 7 | Venlafaxine (150 mg) |
| 8 | Fluoxetine (20 mg), divalproex sodium (500 mg), topiramate (25 mg), modafinil (200 mg) |
| **Study Drop-Outs** | |
| 1 | Sertraline (25 mg), bupropion (100 mg) |
| 2 | Fluoxetine (40 mg) |
| 3 | Paroxetine (30 mg) |
| 4 | Armorthyroid (100 mg) |

## Statistical Analysis

Statistical analyses were performed using SPSS software version 17.0 (SPSS Inc., Chicago, IL). All statistical tests were two-tailed and conducted at the 0.05 significance level. First, two-group analysis of variance (ANOVA) was used to test for potential differences between treatment completers (n = 8) and treatment drop-outs (n = 4) for baseline demographic, clinical, and the endocrine variables (Matsuda Index, TG/HDL ratio, and BMI). Next, repeated-measures ANOVA was conducted to examine changes in depressive severity scores (HDRS-21 and CGI-S) across the 12 weeks of treatment (including week 6 scores) in response to rosiglitazone treatment. Repeated-measures ANOVA also tested for significant changes in the endocrine variables across the 12-week trial. Cohen's d and effect sizes *r* values were calculated where repeated-measures ANOVA showed significance. Lastly, Pearson correlations were used to explore potential associations between the baseline clinical scores and endocrine variables, and between changes in the depressive severity scores and changes in the endocrine variables.

## RESULTS

A total of 15 patients consented to undergo screening for study treatment. Three patients did not meet the minimum FPG or TG/HDL entry criteria, and were thus excluded from the study. The remaining 12 patients met the FPG criteria (n = 5) or TG/HDL criteria (n = 7) and entered the study. Clinical and demographic characteristics for all 12 patients are presented in Table 2. All subjects had a clinical history of multiple depressive episodes beginning in adolescence or in their early 20s. Four patients subsequently dropped out of the study and did not complete the course of 12-week treatment: three due to scheduling conflicts and one due to side effects associated with rosiglitazone (weight gain). No significant differences were noted between completers (n = 8) and drop-outs (n = 4) with respect to age, gender, diagnosis, FPG, TG/HDL ratio, or BMI. However, drop-outs had significantly lower depression severity scores (see Table 2).

**TABLE 2**
**Baseline Characteristics**

| | Completers (n = 8) | Drop-Outs (n = 4) |
|---|---|---|
| | Mean (SD) | |
| Age (years) | 51.9 (5.6) | 52.0 (6.5) |
| BMI ($kg/m^2$) | 34.8 (7.3) | 30.8 (2.6) |
| HDRS-21 | 19.9 (5.0) | 13.0 (0.8)* |
| CGI-S | 4.1 (0.6) | 3.3 (0.5)* |
| FPG (mg/dl) | 101.2 (14.2) | 100.7 (6.2) |
| TG/HDL ratio | 3.01 (2.2) | 2.61 (0.72) |
| Matsuda Index | 2.365 (0.724) | 3.147 (1.910) |
| Entry Criteria Met: | Five FPG, three TG/HDL ratio | Four TG/HDL ratio |
| Gender | Seven females, one male | Four females |
| Diagnosis | Six unipolar, two bipolar | Four unipolar |

* $p < 0.05$.

Repeated-measures ANOVA on HDRS-21 data showed a significant decline from a baseline mean of 19.9 to a post-treatment mean of 12.1 [$F(2,14) = 9.103$, $p = 0.019$]. CGI-S also declined significantly from a baseline mean of 4.0 to a post-treatment mean of 2.9 [$F(2,14) = 7.273$, $p = 0.043$]. The trajectory of HDSR-21 and CGI-S score changes are displayed in Fig. 1. Cohen's d for pre- to post-treatment HDRS-21 was 1.17, with an effect size of $r = 0.504$. For CGI-S scores, Cohen's d and effect size $r$ for CGI-S were 1.27 and 0.54, respectively. Patients showed a mean 39.3% reduction in HDRS-21 scores, with four of the eight patients showing ≥50% score reduction. These same four patients had 12-week HDRS-21 scores well below the HDRS-21 cut-off of ≥12 for subsyndromal depression, with a mean 5.5 at the 12-week evaluation. One subject showed 48% reduction in HDRS-21 score and a 12-week score of 12. The HDRS-21 scores of the remaining three subjects changed in a range from a 27% decrease to an 11% increase, thus indicating nonresponse. Even considering the very small sample size, the trajectories of decline in clinical scores was linear (see Fig. 1). There were no gender differences noted in the results.

Matsuda Index scores declined at a trend level [$F(1,7) = 5.423$, $p = 0.053$), suggesting improvement of IR. Neither BMI nor TG/HDL ratio notably changed; $F(1,7) = 3.541$, $p = 0.102$ and $F(1,7) = 0.127$, $p$ = NS, respectively (see Fig. 1 and Table 3).

Among the 12 patients evaluated at baseline, there were no significant correlations between the depressive severity scores and the endocrine variables, with the exception of a correlation between TG/HDL ratio and HDRS-21 score ($r = 0.619$, $p = 0.042$). This finding remained significant upon an exploratory multiple regression analysis controlling for BMI ($t = 2.739$, $p = 0.026$). Changes in depressive severity scores were not significantly correlated with changes in the endocrine variables, nor were there any correlations between overall percent change in depression severity scale scores and the endocrine variables.

## DISCUSSION

This is the first report, to our knowledge, on the potential utilization of PPAR-agonist rosiglitazone in the treatment of depressed IR patients. An association between IR and affective disorders has been previously postulated by our group[33,34] and others[35,36], but no previous data exist on the use of an insulin-sensitizing agent, such as a PPARγ-agonist, in patients with depression. We previously described a single case of treatment-resistant depression in a woman with PCOS, whose depression remitted after treatment

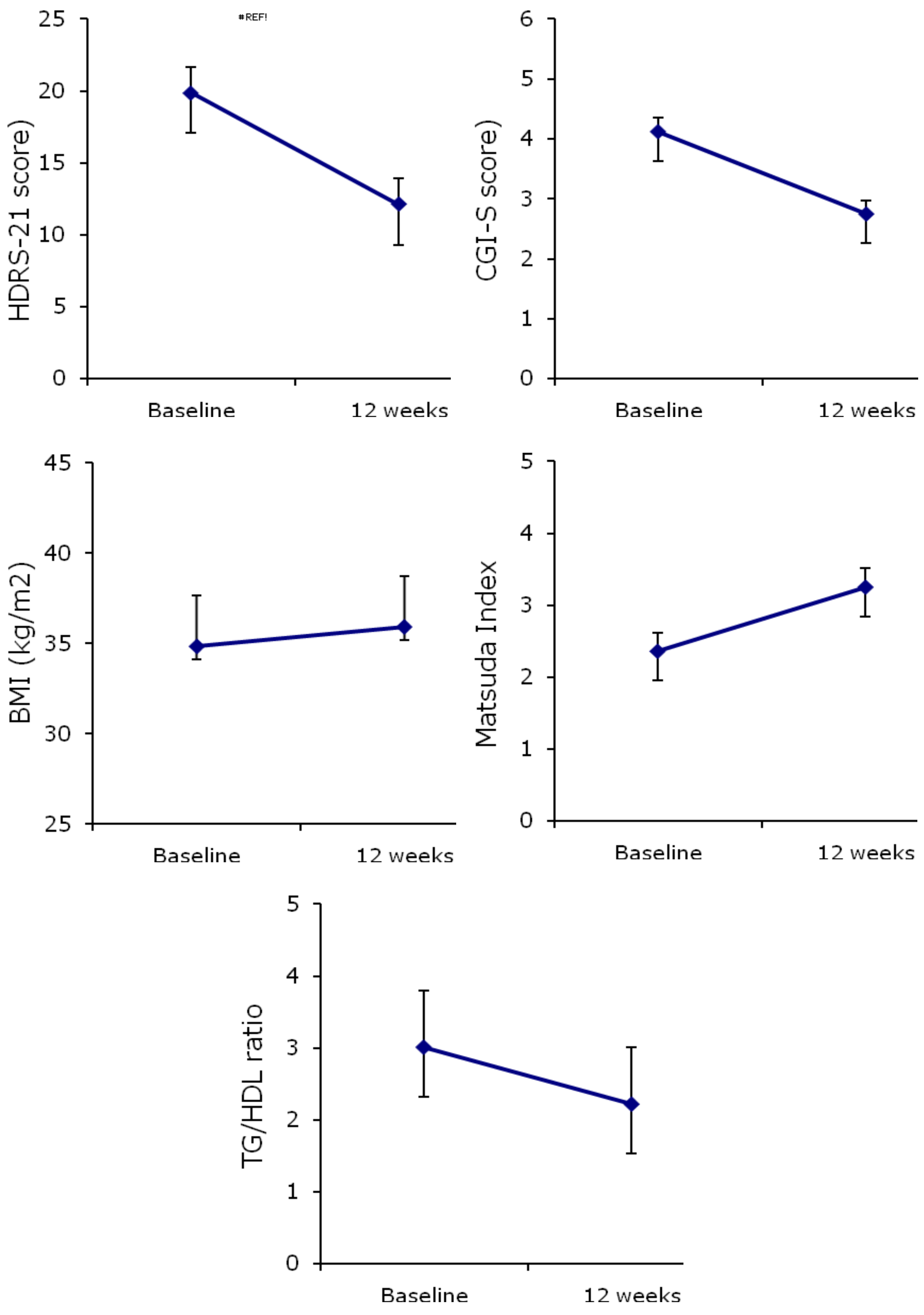

**FIGURE 1.** Depression severity scores (HDRS-21 and CGI-S) improved significantly: $F(2,14) = 9.103$, $p = 0.019$ and $F(2,14) = 7.273$, $p = 0.043$, respectively. The Matsuda Index improved at a trend level [$F(1,7) = 5.423$, $p = 0.053$], while BMI and TG/HDL ratio did not change significantly.

**TABLE 3**
**Pre- and Post-Treatment Matsuda Index, BMI, and TG/HDL Ratios Among Study Completers (n = 8)**

| | Baseline | Post-Treatment (12 Weeks) |
|---|---|---|
| | Mean (SD) | |
| Matsuda Index | 2.37 (0.72) | 3.24 (1.15) |
| BMI (kg/m$^2$) | 35.9 (8.0) | 34.8 (7.3) |
| TG/HDL ratio | 3.01 (2.2) | 2.2 (2.0) |

with metformin (an insulin-lowering medication). The current study sought to evaluate both mood and endocrine changes on treatment with rosiglitazone in patients with depressive disorders. Six of the eight treatment completers had unipolar depression, while two subjects had bipolar depression. There were no differences observed in the HDRS and CGI responses to rosiglitazone, nor in any clinical/demographic characteristics between unipolar and bipolar depressed patients. Interestingly, although we were able to document a degree of improvement in IR per the Matsuda Index after 12 weeks of rosiglitazone treatment, there were no correlations between changes in the depression severity scores and changes in the endocrine variables. This potential uncoupling of the mood improvement from the metabolic effects of rosiglitazone could be type 1 error, or could represent an independent effect of rosiglitazone on mood. We note that the duration of treatment may not have been sufficient in order to evaluate changes in depression severity nor endocrine function in all subjects, and as a pilot it was not powered to examine the effects of rosiglitazone on these variables differentially. The study was also limited by the small sample size and the open-label design. However, our statistical approach was conservative and the effect sizes were of moderate size, even given the small number. These results suggest the potential novel use for an insulin-sensitizing agent in the treatment of depressive disorders. Larger placebo-controlled studies are warranted.

## ACKNOWLEDGMENTS

This study was supported in part by grant M01 RR-00070 from the National Center for Research Resources, National Institutes of Health.

## DISCLOSURE

Dr. Schatzberg has consulted to GSK, Eli Lilly, Wyeth, Forest Laboratories and Pfizer and has equity in Corcept Therapeutics, Forest Laboratories and Pfizer.
